# Colchicine in Contemporary Pharmacotherapy: Mechanistic Insights and Clinical Horizons

**DOI:** 10.3390/jcm14197078

**Published:** 2025-10-07

**Authors:** Łukasz Wołowiec, Joanna Osiak-Gwiazdowska, Albert Jaśniak, Weronika Mucha, Małgorzata Wojtaluk, Weronika Czerniecka, Anna Wołowiec, Joanna Banach, Grzegorz Grześk

**Affiliations:** 1 Department of Cardiology and Clinical Pharmacology, Faculty of Health Sciences, Collegium Medicum in Bydgoszcz, Nicolaus Copernicus University, 87-100 Toruń, Poland; asia.osiak00@gmail.com (J.O.-G.); albertjasniak@gmail.com (A.J.); 312482@stud.umk.pl (W.M.); 312403@o365.stud.umk.pl (M.W.); weronikaczerniecka@interia.pl (W.C.); joannna@op.pl (J.B.); g.grzesk@cm.umk.pl (G.G.); 2 Department of Geriatrics, Division of Biochemistry and Biogerontology, Collegium Medicum in Bydgoszcz, Nicolaus Copernicus University, 87-100 Toruń, Poland; anna.wolowiec@cm.umk.pl

**Keywords:** colchicine, anti-inflammatory agents, cardiovascular disease, dermatologic conditions, COVID-19, neutrophil function, NLRP3 inflammasome inhibition, cytokine release syndrome

## Abstract

Colchicine, a natural alkaloid, has emerged as a promising anti-inflammatory therapy with applications in cardiovascular diseases, dermatological conditions, and COVID-19 management. Its mechanisms, including the modulation of neutrophil activity, the inhibition of the NLRP3 inflammasome, and the mitigation of cytokine storms, have expanded its therapeutic potential beyond traditional uses. This review synthesizes current evidence on colchicine’s clinical applications and mechanisms of action. In cardiovascular medicine, colchicine has been shown to reduce risks of pericarditis, coronary artery disease and atrial fibrillation. In dermatology, most evidence derives from small-scale studies, case series, and retrospective analyses, suggesting potential benefits in conditions such as leukocytoclastic vasculitis, cutaneous amyloidosis, and pemphigus, although these findings require confirmation through randomized controlled trials (RCTs). Emerging studies also indicate a potential role for colchicine in improving outcomes in severe COVID-19. Overall, colchicine demonstrates broad therapeutic utility, warranting further research to clarify its effectiveness across diverse clinical settings.

## 1. Introduction

Colchicine is a fat-soluble alkaloid obtained mainly from species of Colchicum [1]. It was first used in the 6th century by the Byzantine physician Alexander of Tralles for the treatment of gout [2]. Between then and the 13th century, its use was sporadic, primarily for various forms of joint inflammation [3]. Today, colchicine is most commonly used for gout and familial Mediterranean fever [4].

The exact mechanism of colchicine is not fully understood, but its anti-inflammatory effects are largely attributed to binding to tubulin heterodimers, which prevents microtubule polymerization and destabilizes existing microtubules [5]. This disruption interferes with microtubule-dependent processes such as mitosis, intracellular transport, and leukocyte motility, linking its molecular action directly to its clinical anti-inflammatory effects.

Despite its therapeutic value, colchicine has important clinical limitations. Its narrow therapeutic range increases the risk of toxicity, particularly in patients with kidney or liver dysfunction. Additionally, as a substrate of CYP3A4 and P-glycoprotein, plasma concentrations may rise dangerously when combined with inhibitors of these pathways, necessitating careful consideration in clinical practice [4,6,7].

In recent years, colchicine’s unique anti-inflammatory mechanism has attracted growing attention, prompting numerous studies that associate its molecular effects with improved outcomes in cardiovascular diseases, dermatology, and COVID-19 [8]. This review summarizes both the mechanistic basis and expanding clinical applications of colchicine.

This study is a narrative review. Literature was searched for primarily in PubMed, Scopus, and Google Scholar, focusing on recent publications. Review articles, clinical studies, and case reports on the mechanisms of action and clinical applications of colchicine were included, while irrelevant papers were excluded.

## 2. Pharmacokinetics of Colchicine

Colchicine demonstrates dose-dependent effects, and most adverse reactions can be alleviated by dose reduction or discontinuing treatment. Although older regimens used up to 4.8 mg over 6 h for acute gout in patients without renal impairment, current recommendations favor lower doses (1.0–1.2 mg initially, followed by 0.5–0.6 mg after one hour), which provide similar plasma concentrations with better tolerance. [9] Colchicine is primarily absorbed in the small intestine and ileum. After oral administration, it reaches peak plasma concentration within an hour, with a bioavailability ranging from 24 to 88% of the administered dose [10]. Pharmacokinetics show a rapid distribution phase (t1/2 ≈ 1.8 h) followed by a prolonged elimination phase (t1/2 ≈ 20 h) [11]. The most relevant proteins in colchicine pharmacokinetics are P-glycoprotein and CYP3A4 [12]. Colchicine is primarily metabolized by CYP3A4 into 2- and 3-desmethylcolchicine. P-glycoprotein, acting as an efflux pump, influences its absorption in the gastrointestinal tract [10]. Colchicine is primarily excreted through the kidneys. In patients with liver impairment or renal dysfunction, side effects may be more pronounced because these organs face increased strain when metabolizing and eliminating the drug [2]. Colchicine commonly elevates the risk of experiencing diarrhea. This gastrointestinal issue is thought to be linked to the drug’s mechanism, which may promote prostaglandin synthesis, increase intestinal fluid secretion, and stimulate gastrointestinal motility. Other gastrointestinal symptoms, such as nausea and abdominal discomfort are also common [13].

## 3. Colchicine in Cardiovascular Diseases

Inflammation is a key factor contributing to the development of many cardiovascular diseases such as atherosclerosis and coronary artery disease. Numerous epidemiological and clinical studies have consistently shown robust correlations between inflammatory markers and the likelihood of future cardiovascular events. By targeting inflammatory pathways and reducing the immune response, colchicine can alleviate inflammation and improve outcomes in patients with cardiovascular conditions, making it a prominent drug with broad usage in cardiology. This drug is now widely accepted as the standard of care for pericarditis, and multiple clinical trials have explored its effectiveness in treating coronary artery disease, postoperative and post-ablation atrial fibrillation, post-pericardiotomy syndrome, myocarditis and percutaneous coronary interventions.

### 3.1. Acute and Recurrent Pericarditis

Pericarditis is an inflammation of the pericardium, thin sac-like membrane surrounding the heart. It is often caused by a viral infection or heart attack and is characterized by sharp chest pain that may radiate to the left shoulder and neck. Pericarditis accounts for 5% of emergency room visits due to chest pain and 0.1% of hospital admissions [14].

The first documented use of colchicine for pericarditis was reported in 1987 by Rodriguez de la Serna et al. in Barcelona, Spain [15]. They proposed the use of colchicine for pericarditis, based on its efficacy in preventing polyserositis in patients with familial Mediterranean fever. Three years later, Guindo et al. in 1990 [16] from the same facility reported an open-label prospective study on this topic. They treated nine patients with recurrent pericarditis who had experienced multiple relapses despite treatment with other medications. Colchicine was administered at a dose of 1 mg/day, with no recurrence in any patient during a mean follow-up of 24.3 months [17]. Over the years, colchicine has been the subject of many clinical studies on its effectiveness in pericarditis treatment. The Investigation on Colchicine for Acute Pericarditis (ICAP) trial is the largest randomized trial to examine the treatment of pericarditis with colchicine. It was published in 2013 in the New England Journal of Medicine and a total of 240 Italian patients were enrolled. The study found that colchicine reduced symptom persistence at 72 h (19.2% vs. 40.0%, *p* = 0.001), the number of recurrences per patient (0.21 vs. 0.52, *p* = 0.001), and hospitalization rate (5.0% vs. 14.2%, *p* = 0.02). Colchicine also improved the remission rate at 1 week (85.0% vs. 58.3%, *p* < 0.001) with similar adverse effects and drug discontinuation rates in both groups, indicating the potential benefits of using colchicine in reducing recurrent episodes of pericarditis [18]. Another well-known study on this topic was COPE (Colchicine for acute Pericarditis Trial), which included 120 patients that experienced their first episode of acute pericarditis. The mean age of the patients was 56.9 ± 18.8 years, with 54 males included in the study. They were randomized into two groups: conventional treatment with aspirin alone (Group I, *n* = 60) and aspirin plus colchicine (Group II, *n* = 60). The recurrence rate at 18 months was 32.3% in Group I compared to 10.7% in Group II, showing a significant reduction in recurrence with the addition of colchicine. The number of patients requiring treatment to prevent recurrence was 5 (95% CI 3.1 to 10.0). Therefore, this study concluded that colchicine as an adjunct to conventional therapy could be considered a first-choice treatment for acute pericarditis [19].

After many other successful studies [20,21,22], The European Society of Cardiology (ESC) guidelines started to recommend colchicine as a first-line treatment for both acute and recurrent pericarditis, giving it a class I recommendation and level of evidence A. It can be used alongside conventional anti-inflammatory regimens such as aspirin or nonsteroidal anti-inflammatory drugs [14].

### 3.2. Coronary Artery Disease

Coronary artery disease (CAD) is characterized by atherosclerotic plaque accumulation within coronary arteries, leading to reduced myocardial blood flow. The etiology involves complex interactions among genetic predisposition, environmental factors, and lifestyle choices, with endothelial dysfunction playing a central role in atherosclerosis initiation and progression.

One of the first significant global studies on the use of colchicine in the treatment of coronary artery disease (CAD) was published in 2013 and then continued and confirmed in 2020. The study led by Nidorf et al. [23], titled LoDoCo and LoDoCo2, showed that low-dose colchicine (0.5 mg per day) significantly reduced the risk of cardiovascular events in patients with CAD. The studies included more than 5000 patients and showed that colchicine can reduce the risk of heart attacks, strokes, and the need for coronary revascularization procedures [23]. Moreover, the exploratory analysis of this study demonstrates that in patients with chronic coronary artery disease, prolonged anti-inflammatory therapy with low-dose colchicine consistently reduced the incidence of major cardiovascular events across each year during the 5-year follow-up period. These findings reinforce the hypothesis that subclinical vascular inflammation is a persistent process and support the view that effective modulation of the innate immune system requires sustained, long-term treatment rather than short-term intervention [24].

Another study, the COPS (Colchicine in Patients with Acute Coronary Syndrome) trial, was a clinical study aimed at investigating the effectiveness of colchicine in patients with coronary artery disease (CAD), specifically those who had experienced acute coronary syndrome (ACS). It was also published in 2020 and was a randomized, double-blind, placebo-controlled study that involved 17 hospitals in Australia. The results of COPS differed significantly from other trials, showing no statistically significant reduction in the primary composite endpoint (HR 0.65, 95% CI 0.38–1.09, *p* = 0.10). However, when all-cause mortality was replaced with cardiovascular mortality, the colchicine group showed lower incidence (5.0% vs. 9.5%; HR 0.51, 95% CI 0.29–0.89, *p* = 0.019). Notably, COPS demonstrated a concerning mortality signal, with higher rates of non-cardiovascular death (5 vs. 0 deaths, *p* = 0.023) and total death (8 vs. 1, *p* = 0.018) in the colchicine group, though these differences were not statistically significant [25].

The COLCOT study, the third-largest investigation on this topic, was published in 2019. Similarly to its predecessor, COLCOT was a randomized, double-blind, placebo-controlled study that included 4745 patients from multiple countries. The participants also received a daily dose of 0.5 mg of colchicine in addition to standard post-myocardial infarction therapy. All participants had experienced a myocardial infarction within 30 days prior to enrollment in the study. The primary aim of COLCOT was to evaluate whether colchicine administration could reduce the incidence of major cardiovascular events in this patient population. The study demonstrated a statistically significant reduction in the primary composite endpoint (5.5% vs. 7.1%; HR 0.77, 95% CI 0.61–0.96, *p* = 0.02), representing an absolute risk reduction of 1.6% and NNT of 63. The benefit was primarily driven by reductions in stroke (HR 0.26, 95% CI 0.10–0.70) and urgent hospitalizations for angina requiring coronary revascularization (HR 0.50, 95% CI 0.31–0.81) [26].

The largest meta-analysis examining the effect of colchicine on patients with CAD includes all three studies mentioned above. This meta-analysis focused on evaluating the effect of colchicine on major adverse cardiovascular events (MACE) such as myocardial infarction, stroke, and cardiovascular death in adults with coronary artery disease. Twelve randomized controlled trials were included in the meta-analysis, with a total of 13,073 patients (colchicine *n* = 6351, placebo *n* = 6722). The results showed that colchicine significantly reduced the risk of major cardiovascular events, in particular the incidence of myocardial infarction and ischemic stroke, and overall improved cardiovascular outcomes in patients treated with colchicine compared with placebo. However, the study found that the drug may be associated with an increased risk of gastrointestinal side effects and, in some studies, a possible increase in non-cardiovascular mortality, raising concerns about the safety of this therapy [27].

In June 2023, colchicine (0.5 mg daily) received approval from the U.S. Food and Drug Administration for “reduction of atherosclerotic cardiovascular events” in adult patients with established atherosclerotic disease or multiple cardiovascular risk factors. This approval specifically targets the reduction of myocardial infarction, stroke, coronary revascularization, and cardiovascular death in eligible patients. The 2024 European Society of Cardiology (ESC) Guidelines for the management of chronic coronary syndromes provide colchicine a Class IIa recommendation (Level of Evidence A) for patients with atherosclerotic coronary artery disease to reduce myocardial infarction, stroke, and the need for revascularization. The guidelines specify that this recommendation applies to patients with stable coronary artery disease who remain at elevated residual risk despite optimal guideline-directed medical therapy.

A recent study, published in 2024 in the European Heart Journal, investigates the long-term effects of low-dose colchicine in individuals with atherosclerosis. The findings were derived from systematic reviews of case reports, drug registries, and placebo-controlled trials. The study concludes that colchicine is safe for extended use, without causing renal or hepatic dysfunction, nor adversely impacting cognitive function, fertility, or neonatal health. Furthermore, its use does not elevate the risk of cancer, serious infections, or mortality related to specific causes. The primary adverse effect observed is mild diarrhea, which typically resolves within one week of initiating therapy. However, colchicine should be avoided in patients with renal or hepatic impairment, particularly when used in conjunction with CYP3A4 inhibitors or P-glycoprotein transport inhibitors, such as ketoconazole, cyclosporine, or ritonavir, as these combinations may heighten the risk of toxicity. Additionally, the study demonstrates that colchicine does not increase the incidence of myopathy, including rhabdomyolysis, even in the context of statin therapy, which further reinforces the safety and utility of this drug [28].

### 3.3. Atrial Fibrillation

Atrial fibrillation (AF) is the most prevalent sustained cardiac arrhythmia, characterized by a rapid and irregular atrial rhythm resulting from disorganized electrical activity. This condition leads to desynchronization between the atrial and ventricular rhythms, which can manifest as palpitations, shortness of breath, fatigue, and dizziness. Furthermore, AF is associated with a heightened risk of stroke, heart failure, and other cardiovascular complications, primarily due to blood stasis in the atria. The prevalence of AF increases with age and is frequently associated with comorbid cardiovascular conditions such as hypertension, coronary artery disease, and valvular heart disease. Recent research has explored the use of colchicine in the management of AF, especially in cases of atrial fibrillation induced by cardiac surgery and recurrent AF after ablation.

The COPPS-2 study focused on evaluating the use of colchicine in preventing atrial fibrillation (AF) and post-pericardiotomy syndrome (PPS) in patients undergoing cardiac surgery. Colchicine was administered at a dose of 0.5 mg once or twice daily, depending on weight, for 48–72 h before surgery and for one month following cardiac surgery. The study demonstrated mixed results with clear benefits for some endpoints but not others. While colchicine significantly reduced the risk of post-pericardiotomy syndrome (19.4% vs. 29.4%, absolute difference 10.0%, 95% CI 1.1–18.7%, NNT = 10), it showed no statistically significant effect on the primary prevention of postoperative atrial fibrillation in the intention-to-treat analysis (33.9% vs. 41.7%, absolute difference 7.8%, 95% CI −2.2% to 17.6%). However, in the prespecified on-treatment analysis, there was a reduction in postoperative AF (27.0% vs. 41.2%, absolute difference 14.2%, 95% CI 3.3–24.7%, NNT = 7). The trial was associated with a statistically significant increase in gastrointestinal adverse effects (20.0% vs. 11.7%, absolute difference 8.3%, 95% CI 0.76–15.9%, number needed to harm = 12) [29]. However, there are many studies indicating a potential positive effect of colchicine administration after cardiac surgery. One of them was published in 2012, and was focused on the prevention of early recurrence of atrial fibrillation after pulmonary vein isolation [30]. Inflammatory processes induced by the ablation procedure have been associated with the recurrence of the AF [31]. The frequency of atrial fibrillation recurrence was approximately 2.10 times lower in patients taking colchicine compared to those taking placebo. The inclusion of the drug also resulted in a reduction in CRP and IL-6 levels. This study indicates a potentially positive effect of colchicine use in patients after pulmonary vein isolation on preventing arrhythmia recurrence, even without the use of antiarrhythmic drugs [30].

A meta-analysis examining postoperative atrial fibrillation (AF) prevention was published in 2022 and incorporated data from 12 randomized controlled trials (RCTs), encompassing a total of 2274 patients. Among these, 1141 patients were administered colchicine, while 1133 received either a placebo or standard treatment. The analysis demonstrated that perioperative administration of colchicine to individuals undergoing cardiac surgery was associated with a statistically significant reduction in the recurrence of arrhythmias compared to the placebo group. However, additional research is required to optimize the dosing regimen and mitigate the potential risk of adverse effects [32].

Colchicine has also been investigated for preventing AF in non-cardiac settings. The COP-AF study was conducted in 45 centers in 11 countries. Patients took colchicine at a dose of 0.5 mg twice daily for 4 h before the procedure and continued treatment for 10 days. Compared with the placebo group, colchicine unfortunately did not significantly reduce the occurrence of AF and MINS (myocardial injury after noncardiac surgery), but patients more often experienced gastrointestinal symptoms [33].

A systematic review and meta-analysis published in Future Cardiology in 2020 provides key evidence supporting the role of colchicine in reducing major cardiovascular events. The aim of this study was to assess the efficacy of colchicine in patients with cardiovascular diseases. A systematic review and meta-analysis of randomized controlled trials (RCTs) collected up to February 2021 that assessed the risk of cardiovascular events was performed. Data were collected from various databases, including MEDLINE, PubMed, and EMBASE, and 21 studies with a total of 15,569 patients were included in the analysis. The results underline the ability of colchicine to significantly reduce the risk of recurrent pericarditis, major adverse cardiovascular events (MACE), and recurrence of atrial fibrillation, thus increasing its therapeutic potential in cardiology care. Importantly, this study highlights the efficacy of colchicine in preventing the occurrence of myocardial infarction, stroke, cardiovascular death, coronary revascularization, and hospitalization in patients with coronary artery disease, while demonstrating its safety and lack of serious side effects. These data offer valuable insights into the broader use of colchicine in cardiology, positioning it as a promising adjunctive therapy in the treatment of inflammation-induced cardiovascular diseases [34].

### 3.4. Myocarditis

Animal study conducted on mice in 2022 provided some valuable insights into the mechanisms of action of colchicine in acute myocardial infarction (AMI). It demonstrated that the anti-inflammatory properties of colchicine could have a positive impact on survival, cardiac function, and cardiac remodeling after AMI. Administration of colchicine inhibited the formation of neutrophil extracellular traps (NETs) and inflammation following acute myocardial infarction. This inhibition was attributed to reduced NOX2/ROS production and Ca^2+^ influx into neutrophils. Although these preclinical findings are promising, their translation into clinical practice still requires confirmation in human studies [35].

Clinical evidence supporting the beneficial effects of colchicine in patients with myocarditis remains limited. A 2023 case report described two patients with dentate myocarditis who were successfully treated with colchicine at a dose of 0.5 mg daily, in combination with ibuprofen and, in one case, propranolol for tachycardia. Clinical improvement was observed after the introduction of colchicine, and four months of treatment resulted in recovery without complications. However, dentate myocarditis is a specific inflammatory condition, distinct from viral or autoimmune myocarditis [36].

The current evidence base for colchicine in myocarditis is insufficient to support definitive clinical recommendations. Future research should focus on systematic evaluation of colchicine’s efficacy and safety specifically in myocarditis populations before broad therapeutic endorsement.

### 3.5. Cardiopulmonary Bypass

Cardiopulmonary bypass (CPB) is a medical procedure that temporarily takes over the function of the heart and lungs during surgery, maintaining the circulation of blood and the oxygen content of the body. This technique is commonly used in open-heart surgeries, such as coronary artery bypass grafting, heart valve repair or replacement, and certain congenital heart defect repairs. Cardiopulmonary bypass is associated with a significant inflammatory response due to factors such as blood contact with non-physiological surfaces, ischemia–reperfusion injury, and surgical trauma. This response can lead to complications like postoperative atrial fibrillation (AF), myocardial injury, and increased morbidity [37]. Colchicine as an anti-inflammatory drug can potentially reduce the incidence of complications following CBP.

In 2020, a study protocol was published investigating the effects of colchicine on patients undergoing cardiopulmonary bypass (CPB). The randomized, placebo-controlled trial included 132 patients classified as “low risk.” Individuals were randomly assigned in a 1:1 ratio to receive 0.5 mg of colchicine daily or placebo, starting three days before the procedures and continuing for five days after surgery. The main studies are twofold: first, to assess the frequency of myocardial use after CPB; and second, the effects of colchicine on biomarkers derived from myocardium and systemic inflammation. This study is expected to provide valuable information on the basic cardioprotective and anti-inflammatory effects of colchicine in the perioperative setting of cardiac surgery using CBP [38] (Table 1).

**Figure 1:**
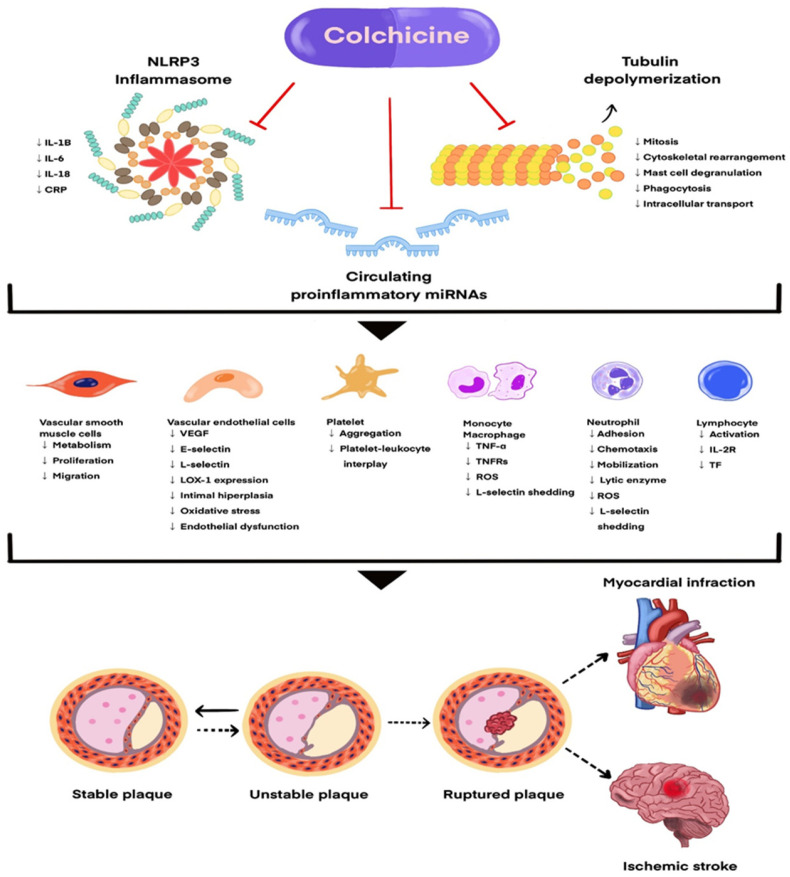
Mechanisms of action of colchicine in inflammation and cardiovascular protection.

The proposed molecular and cellular mechanisms underlying colchicine’s anti-inflammatory and cardioprotective effects are illustrated in Figure 1.

## 4. Colchicine in Dermatologic Diseases

For almost 50 years, colchicine has been continuously studied for potential applications in various complex dermatological conditions. Below, we present about half of the diseases in which colchicine treatment has shown varying degrees of effectiveness. Additionally, the impact of this drug has been investigated on conditions such as psoriasis, neutrophilic eccrine hidradenitis, acne vulgaris, pyoderma gangrenosum, erythema nodosum, erythema elevatum diutinum, erythema induratum, hereditary angioedema, acquired perforating dermatosis, epidermolysis bullosa, dermatitis herpetiformis, and subcorneal pustular dermatosis [39].

### 4.1. Cutaneous Leukocytoclastic vasculitis

Leukocytoclastic vasculitis is a type of small vessel vasculitis that can be found in various forms of vasculitis affecting the skin and internal organs. It is primarily characterized by palpable purpura, and its diagnosis relies on histopathological examination, where the inflammatory infiltrate consists of neutrophils, fibrinoid necrosis, and fragmentation of cell nuclei [40]. Due to these histopathological features, the cutaneous form poses a particular challenge in treatment. Researcher Callen opted for oral colchicine therapy in 13 patients instead of using corticosteroids or immunosuppressants. Among nine patients, colchicine therapy brought complete improvement, partial improvement was observed in three, and one patient showed no effects. However, the challenges did not end there. After discontinuation of colchicine, relapse occurred in seven patients, but upon resuming treatment, control of disease symptoms was restored. Treatment was continued for 2.5 years without signs of toxicity. This long-term treatment highlights the potential of colchicine in managing this condition. This indicates that Callen concludes with fairly good efficacy of this therapy [41]. Plotnick and colleagues presented a case involving a middle-aged woman suffering from leukocytoclastic vasculitis, which did not respond to a daily dose of 60 mg oral prednisone. However, the condition quickly resolved upon the addition of 0.6 mg oral colchicine taken twice daily. Subsequently, a chronic therapy regimen consisting of 5 mg oral prednisone daily alongside 0.6 mg colchicine taken twice daily proved to be sufficient for achieving healing [42]. Unfortunately, in a study conducted by Gemma Seis, colchicine showed no significant therapeutic effect compared to the control group. This suggests variability in patient response to colchicine. In three patients who achieved a full response to colchicine therapy, relapse occurred after treatment cessation, suggesting potential effectiveness in selected patients. Further research is needed to better understand the role of colchicine in treating this disease and to identify predictive factors of treatment effectiveness [43].

### 4.2. Behçet’s Syndrome

Behçet’s disease was first identified in 1937 by the Turkish dermatologist Hulusi Behçet. Initially, He described the condition with a cluster of symptoms: recurrent mouth ulcers, genital sores, and uveitis, which has the potential to cause blindness. Nowadays, it’s understood that Behçet’s disease involves more than this symptom “triad” and can also impact the gastrointestinal tract, central nervous system, and major blood vessels [44]. It is a systemic disease, whose clinical presentation is often very diverse. Most commonly, it follows a course of alternating relapses and remissions [45]. In 1975, it was first observed that colchicine could be effective in treating Behçet’s disease. It is speculated that colchicine exerts its influence on Behçet’s syndrome through leukocyte chemotaxis. The authors noted that administering this substance twice a day for 8–9 months resulted in the resolution of ocular, stomatitis, and joint pain symptoms in 7 out of 12 patients [8,46]. Studies in 2001 on a larger group of patients with Behçet’s syndrome, colchicine was found to be effective in treating certain symptoms of the disease [47]. In a 2009 study involving patients with Behçet’s disease, colchicine demonstrated significant efficacy in improving the overall disease activity index, IBDDAM (Iranian Behçet’s Disease Dynamic Activity Measure), compared to a placebo. The statistically significant differences between the results for colchicine and placebo suggest that colchicine may be an effective treatment for cutaneous and overall symptoms associated with Behçet’s disease [48]. It is worth noting that colchicine is a broad-spectrum anti-inflammatory drug, capable of suppressing the active phase of Behçet’s disease, during which the levels of numerous inflammatory mediators such as IL-1, IL-6, IL-8, IL-12 and TNF-α are elevated [49]. Currently, EULAR recommends colchicine as the first-line treatment for acute arthritis in the course of this syndrome, as well as therapeutic option for isolated mucocutaneous manifestations, particularly in cases of erythema nodosum or genital ulcers when there is no response to topical therapy (level of evidence: Ib; strength of recommendation: A) [50,51].

### 4.3. Epidermolysis Bullosa Acquisita (EBA)

Epidermolysis bullosa acquisita is a rare autoimmune disease in which autoantibodies against collagen VII trigger an inflammatory response, leading to the loss of skin and mucous membrane adhesion. The mechanobullous form of the disease is characterized by skin fragility, blisters, erosions, and milia in areas of trauma. In mild forms of the disease, systemic corticosteroids and immunomodulatory agents such as colchicine and dapsone are effective [52]. In a retrospective study involving 30 patients, colchicine (1.2 mg/day) was considered first-line therapy due to its favorable safety profile; the mean time to remission was 9 months. Patients also received dapsone and prednisone, which increased treatment efficacy [8,53]. Researchers from Japan suggested that the mechanism of action of colchicine in EBA involves the inhibition of antibody secretion, as indicated by the decrease in anti-type VII collagen antibody levels in a patient undergoing treatment. A patient with epidermolysis bullosa acquisita also had type 2 diabetes, and colchicine therapy led to an improvement in hemoglobin A1c levels without any changes in diabetes management [54]. Another group of Japanese researchers described a case of a 65-year-old woman, in whom treatment with a 1 mg daily dose of colchicine yielded better results than the use of steroids [55]. Therefore, colchicine at a dose of 1–2 mg/day, both as monotherapy and in combination with systemic corticosteroids, can prove to be an effective means of controlling mild acquired EBA. With minimal side effects such as diarrhea, this therapeutic option is beneficial, especially for patients who should avoid immunosuppressants. Overall, the mechanisms of colchicine action, involving the inhibition of neutrophil chemotaxis and an increase in prostaglandin E2 production, make it a favorable choice in the treatment of EBA [52,54].

### 4.4. Pemphigus

Pemphigus is a clinically and epidemiologically heterogeneous group of autoimmune blistering diseases, including pemphigus vulgaris, pemphigus foliaceus, paraneoplastic pemphigus, IgA pemphigus, and pemphigus herpetiformis. IgA pemphigus of the subcorneal pustular dermatosis (SPD) type is distinguished by the presence of subcorneal acantholysis and a high concentration of neutrophils [56]. In 1999, an Israeli group reported treating two cases of IgA pemphigoid using 0.5 mg of colchicine three times a day, resulting in symptom resolution within two weeks. Recurrences occurred each time colchicine was discontinued [57]. In the rare and difficult-to-treat variant of pemphigus—pemphigus foliaceus, researchers from Japan achieved a 6-month remission period using colchicine at a dose of 0.5 mg three times a day and prednisone (steroid) at a dose of 30 mg daily led to gradual regression of skin lesions, highlighting its potential role in refractory cases [58]. However, further studies with larger patient populations are required to definitively confirm the efficacy and safety of colchicine in dermatologic conditions.

### 4.5. Mucous Membrane Pemphigoid (MMP)

Lesions in the oral cavity and on the conjunctivae are most commonly seen in MMP, a pemphigoid disease with predominant mucosal involvement. Outside the mouth, these lesions may scar, leading to severe issues with vision, breathing, and food intake [59]. A Greek research group reported colchicine efficacy in 67% patients, surpassing other therapies such as dapsone in the treatment of skin lesions [60]. In line with these findings, a retrospective study conducted in Bordeaux demonstrated that colchicine may serve as an effective second-line therapy in patients with refractory MMP, achieving remission of oral lesions in over 80% of cases, with better tolerance compared to dapsone. Despite these encouraging results, disease relapses still occur, underscoring the need to further research of optimal therapeutic strategies [61].

### 4.6. Pigmented Purpuric Dermatoses (PPD)

Pigmented purpuric dermatoses represented a group of chronic skin disorders marked by petechiae, purpura, and pigmentary alterations. They usually present as reddish-brown patches with a distinctive “cayenne pepper” pattern, most often seen on the lower legs. Subtypes include Schamberg’s disease, Majochi’s disease, Gougerot-Blum disease, Ducas and Kapetanakis pigmented purpura, and lichen aureus, with diagnosis based mainly on clinical findings and occasionally supported by biopsy [62]. This benign and chronic condition does not have a gold standard treatment [63]. A case of a previously healthy 32-year-old woman with asymptomatic leg lesions was described. She presented with purpuric skin eruptions and brownish stains dispersed on her lower limbs. Biopsy confirmed the diagnosis of PPD, specifically Schamberg’s disease. Colchicine therapy was initiated, yielding promising clinical outcomes. The patient was subsequently followed up on outpatient visits for 10 months, during which no recurrences were observed [64]. Another research group from India described the case of a 45-year-old man suffering from PPD, which did not respond to topical application of 0.1% betamethasone valerate cream and the use of compression stockings. The disease was cured after 3 months of treatment with oral colchicine at a dose of 1 mg per day, combined with calcium dobesilate at a dose of 1 g per day [65]. The reported cases suggest that colchicine may represent an effective therapeutic option in the management of PPD, particularly in patients unresponsive to standard topical therapies, contributing to lesion resolution and reducing the risk of recurrence.

### 4.7. Sweet’s Syndrome

Sweet’s syndrome is associated with a significant infiltration of neutrophils and increased chemotactic activity of these cells in peripheral blood. The management of this disease is primarily based on systemic corticosteroids, although colchicine, dapsone, and potassium iodide have also been reported as effective alternatives. In addition, the use of conventional immunosuppressive agents, biologic therapies, and selected small molecules has shown beneficial outcomes [66]. Researchers speculate that colchicine in Sweet’s syndrome may act similarly to its action in Behçet’s disease—that is, by inhibiting the chemotaxis of neutrophils, reducing their mobility, blocking adhesion, and stabilizing lysosomal membranes. These mechanisms highlight the broad potential applications of colchicine in inflammatory diseases. A study from 1981 indicated that colchicine may alleviate symptoms of Sweet’s syndrome and be more effective than other medications. [67] The retrospective study of 90 patients revealed that colchicine was the most commonly used treatment, taken by 48.9% of the study population. A dose of 1–2 mg per day was administered to 30 patients (68.2%), with regression of lesions observed within 1–14 days of starting the therapy. Among the 13 patients who initially did not show improvement with colchicine, the addition of prednisone resulted in their improvement [68]. In 2021, researchers from Thailand reported a case of a patient with the bullous variant of Sweet’s syndrome. The therapy involving a combination of colchicine and acitretin was found to be effective [69]. In summary, colchicine may be useful in the treatment of Sweet’s syndrome in patients who do not respond to systemic corticosteroids [39].

### 4.8. Actinic Keratosis

Actinic keratosis is a skin lesion that develops in areas exposed to prolonged UV radiation, most often appearing as a rough, scaly papule. Its presentation can vary widely, from subtle flat spots to thickened lesions, and in some cases it may form a cutaneous horn [70]. Colchicine gel provides another form of application. In a comparative study evaluating its effectiveness against diclofenac gel in treating actinic keratosis, colchicine proved to be a safe and effective treatment, characterized by fewer adverse effects and no recurrence [71]. Additionally, other studies examined the impact of different concentrations of topically applied colchicine on the clinical improvement of the disease, revealing that both 0.5% and 1% concentrations are equally effective in treating actinic keratosis [72]. For now, the most recent guidelines on the management of actinic keratosis do not include colchicine as a therapeutic option [70].

### 4.9. Chronic Urticaria

Chronic urticaria is a skin condition in which hives, swelling or both repeatedly occur for at least six weeks, often causing itching and discomfort [73]. The use of colchicine in the treatment of urticaria remains controversial. In a case–control study conducted by Nabazivadeh et al., the use of colchicine in the treatment of 55 patients was proposed; however, statistically significant improvements among subgroups were not demonstrated, although colchicine proved helpful in alleviating symptoms for patients [74]. In the study regarding the therapy of chronic urticaria resistant to antihistamine treatment, 22 patients participated. Nine of them received colchicine as part of the therapeutic regimen. Among these patients, seven showed complete control of urticaria after 12 weeks of treatment. The study involved regular assessments of patients during the first month and monthly for the next 2 months, with therapy effectiveness evaluated based on the reduction in urticaria score. It is worth noting that colchicine was effective in the majority of patients, suggesting its potential application in the treatment of resistant chronic urticaria [75].

### 4.10. Recurrent Aphthous Stomatitis (RAS)

Recurrent aphthous stomatitis (RAS) is a chronic disorder of the oral mucosa. It is marked by the appearance of small, recurrent ulcers, either solitary or multiple, with an erythematous halo and a yellowish-gray base [76]. A study from 2002 demonstrated that colchicine is an effective preventive treatment for severe aphthous stomatitis. After three months of treatment, 63% of patients experienced improvement, with this effect persisting in 37% of them [77]. A double-blind randomized clinical trial was conducted on 34 patients with RAS. It showed that both prednisolone and colchicine were effective in treating this condition. Considering that both therapies worked similarly and colchicine resulted in more side effects, 5 mg/day of prednisolone appears to be a better alternative in reducing the symptoms of the disease [78]. A retrospective study was conducted between 2012 and 2016 and included 150 patients with recurrent aphthous stomatitis treated with colchicine. A total of 114 patients were eligible for analysis, in whom treatment efficacy, safety and drug survival were assessed. Moderate or significant clinical improvement was observed in more than 80% of patients, while adverse events occurred mainly within the first two weeks of therapy and included gastrointestinal complaints (16.7%), neutropenia (3.5%), and elevated liver enzyme levels (4.4%). Long-term treatment maintenance was more common in patients with milder ulcerative lesions, whereas extensive ulcers were associated with a higher likelihood of early treatment discontinuation [79]. In randomized, prospective clinical trial, the effectiveness of a betamethasone mouth rinse, colchicine tablets, and their combination was evaluated in 106 patients with recurrent aphthous stomatosis. All three treatment approaches resulted in a comparable reduction in lesion severity—approximately 50% after nine months of therapy. No significant differences were observed between the methods. The authors empasized that the greatest benefits appear after at least six months of treatment, and both betamethasone and colchicine may be considered effective options for long-term management of RAS [80].

### 4.11. Urticarial Vasculitis

Urticarial vasculitis is an uncommon autoimmune condition in which edematous papules and plaques persist for more than 24 h and are frequently accompanied by systemic manifestations such as fever and joint pain. Unlike ordinary urticaria, it is driven by inflammation of small blood vessels, producing more severe, long-lasting skin lesions that may resemble bruising [81]. This disease may be associated with viral illnesses, systemic lupus erythematosus, Sjögren’s syndrome, or serum sickness, and its treatment includes various therapies such as antihistamines, prednisone, and immunosuppressive drugs, although they are not always effective. In a study from 1985 by Wiles and colleagues, colchicine was found to be an effective treatment for urticarial vasculitis in 2 patients who had failed other therapies. These patients had previously been treated with antihistamines, immunosuppressants, and prednisone [82]. In another study, the treatment with colchicine yielded results, unlike indomethacin, steroids, dapsone, and hydroxychloroquine. This was described by Asherson and colleagues in 1991 [83]. The conclusions drawn from the retrospective cohort study suggest that colchicine is as effective as corticosteroids as first-line therapy in the treatment of urticarial vasculitis. In patients with recurrent or refractory disease, immunosuppressive drugs appear to be more effective than rituximab, indicating the potential efficacy of colchicine in this disease [84]. Confirmation of the importance of colchicine can also be found in the case of a patient with Sjögren’s syndrome and urticarial vasculitis. She was treated with prednisolone, initially at a dose of 5 mg/day, which was later increased to 35 mg/dl due to worsening symptoms. The addition of colchicine (1 mg/day) enabled effective control of skin lesions and dyspnea and allowed for a rapid tapering of the corticosteroid dose. This therapeutic approach led to significant clinical improvement and a decrease in anti-SSA antibody levels, highlighting the role of colchicine in suppressing neutrophil infiltration and stabilizing disease progression [85]. However, further studies are needed to confirm these findings and establish the optimal therapeutic strategy [84].

### 4.12. Primary Localized Cutaneous Amyloidosis (PLCA)

In primary localized cutaneous amyloidosis (PLCA), various amyloid proteins deposit in the skin without affecting other organs [86]. Subtypes of PLCA include lichen amyloidosis, macular amyloidosis, and nodular amyloidosis [87]. Fifteen individuals diagnosed with PLCA, comprising 7 with lichen amyloidosis and 8 with macular amyloidosis, were administered oral colchicine (1 mg/day) for a duration of 3 months. Among those with macular amyloidosis, all reported complete alleviation of itching, whereas individuals with lichen amyloidosis experienced a notable reduction (30–60%) within just 15 days. By the end of the 90-day treatment period, nearly complete disappearance of pigmentation was observed in those with macular amyloidosis, while individuals with lichen amyloidosis saw a substantial decrease (80–98%) in papule size. Notably, no significant adverse effects were reported as a result of colchicine therapy [88]. A review from 2013 shows a study involving 94 patients, with tumors most commonly occurring in the head and neck area (34%), and two or more lesions were found in different locations in 20 patients (22%). Subsequently, local recurrence occurred in 8 patients (9%), mainly in men with nodular PLCA localized in the facial area. These findings underscore the importance of continued monitoring for recurrence [89].

### 4.13. Cutaneous Sarcoidosis

Cutaneous sarcoidosis typically manifests as pink to reddish-brown papules or plaques, most often located on the head and neck. Since skin lesions are easily accessible for clinical assessment and biopsy, dermatologic evaluation can be valuable in confirming the diagnosis of sarcoidosis [90]. Three patients with facial cutaneous sarcoidosis were reported to have rapidly responded to treatment, achieving long-term remission with a combination therapy of colchicine and topical corticosteroid ointment [91]. Several years later, in another patient with sarcoidosis who had skin symptoms such as papules and erythematous infiltrated plaques on the face, back, shoulders and lower limbs and nodules on elbows, the decision was made to initiate colchicine therapy. The patient also had hepatitis C. In patients with this condition, the use of hepatotoxic drugs such as methotrexate was limited, and prednisone alone, previously administered at recommended doses, had not provided sufficient benefit. Despite the reported therapeutic benefits, there is a need for further studies, particularly randomized controlled trials, to definitively confirm the efficacy of colchicine in cutaneous sarcoidosis [92].

**Figure 2:**
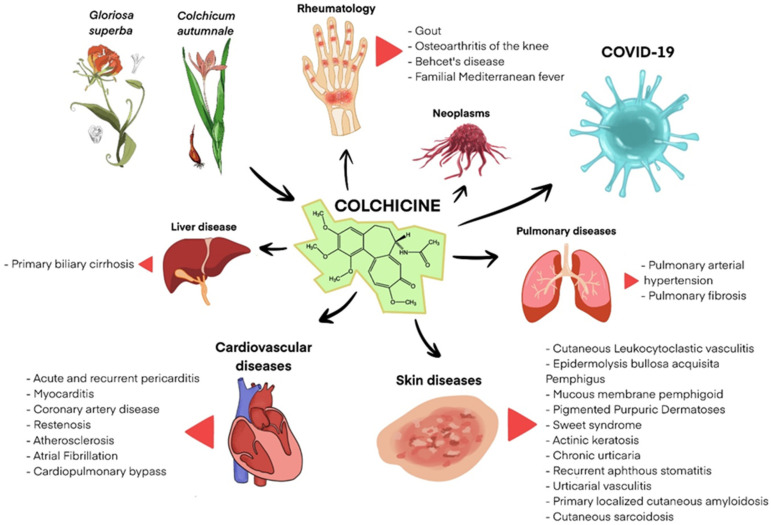
Clinical applications of colchicine.

Below is a table summarizing the effectiveness of colchicine in selected dermatologic conditions discussed in this article (Table 2, Figure 2).

## 5. Colchicine in COVID-19

COVID-19, an infectious respiratory disease, emerged in 2019 in the Chinese city of Wuhan. It is caused by the coronavirus 2, also known as SARS-CoV-2. The pandemic rapidly spread, posing a threat not only to China but to the entire world [93].

The main receptor believed to be involved in the development of COVID-19 infection is ACE2. The location of ACE2 receptors on cardiac muscle cells allows the SARS-CoV-2 virus to penetrate the host cells and directly invade heart tissue. This leads to cardiovascular complications post-infection, such as heart failure, inflammation, or even myocardial infraction [2]. Additionally, patients with this disease often experience a “cytokine storm,” prompting the use of colchicine, known for its anti-inflammatory properties, in the treatment of this condition [94].

One practical advantage of colchicine is its oral administration [95]. One of its main actions is the inhibition of caspase-1 activity, indirectly affecting the NLRP3 inflammasome. NLRP3’s function includes the production of pro-inflammatory cytokines such as IL-1β, which also induces IL-6 production. Blocking NLRP3 leads to a reduction in the release of these mediators, thereby contributing to the decrease in the inflammatory response [95]. Colchicine’s additional anti-inflammatory action involves the inhibition of adhesion molecule expression and lymphocyte recruitment [96].

In recent years, several studies have confirmed the effectiveness of colchicine in SARS-CoV-2 infection. The results of the randomized GRECCO-19 study showed a significant delay in clinical deterioration in patients treated with colchicine (from 14% in the control group to 1.8% in the colchicine group). However, there was no significant difference in the levels of C-reactive protein and cardiac troponin [97].

Furthermore, there have been reports of individuals with gout who, due to chronic colchicine use, exhibited a milder course of SARS-CoV-2 infection. N. Mansouri and others described a case of a 42-year-old patient with monoarticular gout and concomitant COVID-19 infection [98]. Treatment with colchicine resulted in satisfactory outcomes regarding both general health status and inflammatory state improvement.

A randomized controlled trial by Tardif et al. [26] (COLCORONA, 2021) evaluated colchicine in non-hospitalized COVID-19 patients. In the overall study population, the drug did not significantly reduce the composite endpoint of hospitalization or death. In the PCR-confirmed subgroup, a trend toward lower hospitalization risk was observed (RR 0.56; 95% CI 0.19–1.66), but this result was not statistically significant. In contrast, the large RECOVERY trial in hospitalized patients and subsequent meta-analyses consistently showed no reduction in mortality with colchicine. Taken together, the evidence suggests that any potential benefit of colchicine may depend on patient selection and disease stage but remains unproven.

Some smaller studies, however, have suggested possible benefits. Lopes et al. [99] reported that colchicine shortened the duration of COVID-19 symptoms by approximately two days compared with placebo (7 vs. 9 days, *p* = 0.003) [99]. While encouraging, such findings require confirmation in larger randomized trials before firm conclusions can be drawn.

By contrast, the large RECOVERY trial in hospitalized patients (over 11,000 participants) and subsequent meta-analyses consistently showed no reduction in mortality with colchicine. These results suggest that any potential benefit may depend on disease stage, with greater likelihood in the early inflammatory phase [99,100,101,102]. Results from the conducted meta-analysis regarding the use of colchicine in patients with COVID-19 indicate that, despite analyzing 23 randomized controlled trials (RCT) involving 28,249 participants, no statistically significant reduction in mortality risk was observed (RR 0.99; 95% CI 0.93 to 1.05; *p* = 0.78). This suggests that colchicine, despite its anti-inflammatory properties, does not offer significant clinical benefits in the later stages of the disease [99]. Therefore, considering the use of this drug is best done in the early inflammatory phase (phase 2) [95]. Similar conclusions can be drawn from the COLVID19 study, which involved 227 patients, with 152 undergoing analysis. The results did not reveal significant differences in the primary endpoints, such as the need for mechanical ventilation, admission to the intensive care unit, or mortality. In the colchicine group, 14.3% of patients progressed to a critical phase, which was comparable to 13.3% in the control group. The authors of this study suggest that potential reasons for the lack of treatment efficacy may include the administration of colchicine at a stable rather than saturating dose, the variable nature of the disease, and differing clinical characteristics among patients [103].

Mitev, in his study, highlights that insufficient colchicine doses used in many clinical trials may have contributed to the lack of observed efficacy in treating COVID-19. He suggests that lower doses may not reach the minimal micromolar concentrations in leukocytes necessary to inhibit the NLRP3 inflammasome, mitigate the cytokine storm, and prevent severe disease complications. In his research involving 785 hospitalized patients, higher colchicine doses were associated with a reduction in mortality rates. However, these findings stem from a non-randomized study and may be influenced by selection bias. Safety considerations are crucial, as higher colchicine doses approach toxicity thresholds and carry increased risks, especially when combined with CYP3A4 or P-gp inhibitors. Mitev also questions the WHO’s “strong recommendation against colchicine use in COVID-19,” noting that many studies leading to this conclusion employed doses likely insufficient to achieve therapeutic effects. While intriguing, these dose-related observations cannot replace evidence from randomized controlled trials (RCTs). [104].

Taking into consideration the above results, it is essential to remember that despite numerous cases where colchicine has demonstrated its beneficial effects in patients with COVID-19, its impact on this disease still undergoes extensive research. In the future, these studies will provide a more thorough understanding of its advantages and limitations [105]. Table 3 provides a comprehensive summary of colchicine’s mechanisms of action, clinical evidence, challenges, and future perspectives in the context of COVID-19 management.

## 6. Risks and How to Minimize Them

Numerous studies have confirmed the safety of low-dose colchicine; however, it is not devoid of side effects and carries a certain level of toxicity. The most commonly reported side effect in patients receiving colchicine is diarrhea, and at higher doses, toxic effects on the gastrointestinal tract may occur [13]. A major challenge in the clinical use of colchicine is its narrow therapeutic index and plasma toxicity. Therapeutic plasma concentrations range from 0.5 to 3 μg/L, with toxicity symptoms typically emerging at concentrations around 3 μg/L [106].

Colchicine can possibly interact with numerous drugs because it is a substrate for P-glycoprotein and cytochrome CYP3A4. Co-administration of colchicine with CYP3A4 inhibitors or P-gp inhibitors can significantly elevate its plasma concentration, in some cases increasing the risk of toxicity up to fourfold [106]. In patients with hepatic or renal impairment, dosage adjustments are crucial to minimize the risk of toxicity [107].

Symptoms of colchicine poisoning may occur at doses as low as 0.5 mg/kg body weight, with doses exceeding 0.8 mg/kg often proving fatal [108]. The clinical manifestations of poisoning are related to the disruption of cellular mitosis and typically proceed through three phases. The early phase (10–24 h post-ingestion) is characterized by nausea, vomiting, diarrhea, and other gastrointestinal symptoms. The intermediate phase (2–7 days) involves multiple organ failure, disseminated intravascular coagulation (DIC), and cardiovascular collapse. The late phase (after day 7) determines the patient’s outcome, with recovery or death depending on the severity of organ damage. Recovery often includes rebound leukocytosis and alopecia [106,108].

At present, there is no definitive antidote for colchicine poisoning. Current methods are designed to prevent the serious consequences of an overdose, but they cannot reverse the effects of colchicine. A potential antidote currently being studied is Colchicine-Specific Fab Fragments and Lipocalins, which have been shown to bind colchicine and promote its elimination. While these agents have demonstrated efficacy in animal models, they are not yet commercially available for clinical use [109].

## 7. Colchicine in Guidelines

Colchicine has found its place in the latest ESC Guidelines for the management of chronic coronary syndromes 2024. According to the guidelines, patients with chronic coronary syndrome and atherosclerotic coronary artery disease should consider administering a low dose of colchicine (0.5 mg daily) to reduce the incidence of myocardial infarction, stroke—class of recommendations IIa, level A [110]. This recommendation appeared after the results of the COLCOT (Colchicine Cardiovascular Outcomes Trial), LODOCO2 (Low-Dose Colchicine 2) trial and a meta-analysis covering 12,000 patients [23,26,111].

## 8. Discussion

The article highlights the versatile applications of colchicine, which, due to its unique anti-inflammatory properties, has found uses in treating a variety of conditions. While historically associated with gout management, its role has significantly expanded into dermatology, cardiology, and COVID-19 therapy.

In dermatology, colchicine has proven effective in conditions like epidermolysis bullosa acquisita (EBA), Sweet’s syndrome, and Behçet’s disease, where it suppresses inflammatory processes and neutrophil chemotaxis. In vitro studies suggest that colchicine may enhance collagenase activity in certain forms of EBA, reducing excessive collagen deposition and supporting wound healing. These findings pave the way for further research, especially concerning dystrophic forms of EBA and chronic wound complications.

In cardiology, colchicine has emerged as a valuable tool in managing pericarditis, coronary artery disease, and atrial fibrillation. Studies like LoDoCo2 and ICAP demonstrate its ability to reduce the recurrence of pericarditis and mitigate cardiovascular events such as heart attacks and strokes. Its mechanism involves suppressing inflammatory pathways and stabilizing microcirculation. However, its narrow therapeutic index and potential drug interactions, especially in patients with renal or hepatic impairment, necessitate cautious application.

In the context of COVID-19, colchicine has gained attention as a potential therapy to modulate the inflammatory response, particularly in preventing cytokine storms. Studies such as GRECCO-19 and those conducted by Tardif suggest a possible reduction in hospitalization risk and shorter symptom duration. However, the RECOVERY trial failed to confirm these benefits in advanced disease stages, underscoring the need for precise timing and dosing strategies.

## 9. Conclusions

Colchicine, a natural alkaloid with well-documented anti-inflammatory properties, has proven to be a versatile therapeutic agent beyond its traditional use in gout and familial Mediterranean fever. Its efficacy in cardiovascular diseases, including pericarditis, coronary artery disease, and atrial fibrillation, highlights its role in mitigating inflammation and improving patient outcomes. In dermatology, colchicine demonstrates potential in managing conditions such as leukocytoclastic vasculitis, Behçet’s syndrome, and epidermolysis bullosa acquisita. Moreover, its ability to modulate neutrophil activity and suppress the NLRP3 inflammasome offers promising applications in COVID-19 management, particularly in reducing cytokine storms. Despite these benefits, careful consideration of dosage, therapeutic range, and potential drug interactions is crucial due to its narrow safety profile. Further research is necessary to optimize its applications and fully elucidate its long-term safety and efficacy across various clinical domains.

## Figures and Tables

**Table 1:** Presents a comprehensive summary of key clinical studies investigating colchicine in cardiovascular applications.

Trial	Population	Dose	Primary Endpoints	Main Adverse Effects	Conclusions
LoDoCo (2013)	*n* = 532, stable CAD	Col 0.5 mg vs. control	HR 0.33 (0.18–0.59), ARR 10.7%, NNT 9	GI disturbances	Significant benefit
LoDoCo2 (2020)	*n* = 5522, stable CAD	Col 0.5 mg vs. placebo	HR 0.69 (0.57–0.83), ARR 2.8%, NNT 36	GI effects, possible ↑non-CV death	Significant benefit with safety signal
COLCOT (2019)	*n* = 4745, recent MI	Col 0.5 mg vs. placebo	HR 0.77 (0.61–0.96), ARR 1.6%, NNT 63	GI disturbances	Modest but significant benefit
COPS (2020)	*n* = 795, ACS	Col 0.5 mg BID/daily vs. placebo	HR 0.65 (0.38–1.09), *p* = 0.10	↑Non-CV mortality signal	No significant benefit, mortality concerns
COPPS-2 (2014)	*n* = 360, cardiac surgery	Col 0.5 mg vs. placebo	ARR 10.0%, NNT 10	↑GI effects (NNH = 12)	Benefit for PPS, no benefit for POAF

Table demonstrates the heterogeneity in patient populations, with more pronounced benefits observed in stable coronary artery disease populations compared to acute coronary syndromes and highlights the consistent gastrointestinal tolerability concerns across all studies.

**Table 2:** Summary of colchicine effectiveness in selected dermatologic diseases according to quality of evidence.

Disease	Type of Studies/Level of Evidence	Sample Size (*n*)	Effectiveness Assessment and Conclusions (Grade)
Leukocytoclastic Vasculitis	Case reports, series of 13 patients (Callen, 1985) [41]; RCT (Sais, 1995) [43]	single cases; *n* = 13; RCT: *n* = 41	Low—variable efficacy; frequent relapses after discontinuation; one negative RCT
Behçet’s Syndrome	case series; RCTs: (2001,2009);	*n* = 12; RCT: *n* = 116, *n* = 169	Moderate—good result in mucocutaneous and articular manifestations: supported by EULAR recommendations
Epidermolysis Bullosa Acquisita	Retrospective series, case reports	*n* = 30; single cases	Low-effective in mild forms; favorable safety profile; no RCTs
Pemphigus (IgA, foliaceus)	Case reports (2–3 patients)	<5	Very low—only case reports; requires controlled studies
Mucous Membrane Pemphigoid	Retrospective series, observational studies	*n* = 80	Low-moderate—efficacy in oral involvement; better tolerance than dapsone; 67% response in series; >80% remission in retrospective study
Pigmented Purpuric Dermatoses	Case reports	2 patients	Very low—only cases; favorable outcome but no controlled data
Sweet’s Syndrome	retrospective study (*n* = 90); case reports	*n* = 90; single cases	Moderate—effective in most patients; rapid improvement; no RCts, but large series supports use
Actinic Keratosis	RCTs (vs diclofenac, 2016; 0.5% vs. 1%, 2001)	*n* = 70, *n* = 62	Moderate—effective topical therapy, well tolerated; not included in guidelines
Chronic Urticaria	clinical trials	*n* = 55, *n* = 22	Low—partial benefit in some patients; evidence inconsistent
Recurrent Aphthous Stomatitis	RCTs (2002, 2010, 2023); retrospective study	*n* = 54, *n* = 34, *n* = 106; *n* = 150	Moderate—confirmed efficacy; comparable to steroids; side effects limit use
Urticarial Vasculitis	Case reports, retrospective cohort	Single cases; *n* = 57	Low—some patients respond; more effective than NSAIDs; RCTs lacking
Primary Cutaneous Amyloidosis	Small series, systematic review	*n* = 15, *n* = 94	Low—improved pruritus and pigmentation; no controlled trials and
Cutaneous Sarcoidosis	case reports	*n* = 3, *n* = 1	Very low—only case data; possible role as adjunct therapy

**Table 3:** Summary of Colchicine in the Context of COVID-19.

Aspect	Details
Mechanism of Action	- Inhibits microtubule polymerization and reduces inflammation by suppressing NLRP3 inflammasome activity. - Decreases cytokine release (e.g., IL-1β, IL-6). - Limits lymphocyte recruitment and adhesion molecule expression.
	- Oral administration. - Established anti-inflammatory properties. - Evidence of effectiveness in early inflammatory stages of COVID-19.
Clinical Evidence Supporting Use	- **GRECCO-19 Study**: Reduced clinical deterioration rate from 14% (control) to 1.8% (colchicine). - **Tardif et al.** (2019) [26]: Reduced hospitalization risk by ~44%. trend; not statistically significant - **Lopes et al.**: [99] Shortened symptom duration by 2 days (*p* = 0.003). - **Mansouri et al**. [98]: Chronic colchicine use in gout patients linked to milder COVID-19 cases
Challenges and Controversies	- **RECOVERY Trial**: No significant impact on 28-day mortality (11,340 patients). - Meta-analysis (23 RCTs, 28,249 participants): No significant reduction in mortality risk (RR 0.99; *p* = 0.78). - Limited efficacy in advanced disease stages.
Dose-Dependent Considerations	- Mitev study: Insufficient doses in trials may fail to achieve therapeutic leukocyte concentrations for effective inflammasome inhibition. - Higher doses linked to a sevenfold reduction in mortality in hospitalized patients.
Future Perspectives	- Further studies are essential to clarify optimal dosing strategies, therapeutic timing, and patient selection criteria for maximizing colchicine’s benefits in COVID-19 treatment.
